# Role of Oligodendrocytes and Myelin in the Pathophysiology of Autism Spectrum Disorder

**DOI:** 10.3390/brainsci10120951

**Published:** 2020-12-08

**Authors:** Alma Y. Galvez-Contreras, David Zarate-Lopez, Ana L. Torres-Chavez, Oscar Gonzalez-Perez

**Affiliations:** 1 Department of Neuroscience, Centro Universitario de Ciencias de la Salud, University of Guadalajara, Guadalajara 44340, Mexico; 2 Laboratory of Neuroscience, School of Psychology, University of Colima, Colima 28040, Mexico; dzarate@ucol.mx (D.Z.-L.); torres_chavez@ucol.mx (A.L.T.-C.); 3 Physiological Sciences PhD Program, School of Medicine, University of Colima, Colima 28040, Mexico

**Keywords:** oligodendrogenesis, myelination, epidermal growth factor, insulin-like growth factor

## Abstract

Autism Spectrum Disorder (ASD) is an early neurodevelopmental disorder that involves deficits in interpersonal communication, social interaction, and repetitive behaviors. Although ASD pathophysiology is still uncertain, alterations in the abnormal development of the frontal lobe, limbic areas, and putamen generate an imbalance between inhibition and excitation of neuronal activity. Interestingly, recent findings suggest that a disruption in neuronal connectivity is associated with neural alterations in white matter production and myelination in diverse brain regions of patients with ASD. This review is aimed to summarize the most recent evidence that supports the notion that abnormalities in the oligodendrocyte generation and axonal myelination in specific brain regions are involved in the pathophysiology of ASD. Fundamental molecular mediators of these pathological processes are also examined. Determining the role of alterations in oligodendrogenesis and myelination is a fundamental step to understand the pathophysiology of ASD and identify possible therapeutic targets.

## 1. Introduction

In 1943, Kanner described the first symptoms of Autism Spectrum Disorder (ASD) as an innate disturbance of affective contact. In 1944, Hans Asperger included a psychopathic disturbance of social interaction in the symptomatology of ASD [1]. To date, ASD is considered an early neurodevelopmental disorder [1], which is characterized by substantial deficits in social interaction and communication associated with repetitive and restricted behaviors [2]. The incidence of ASD around the world is 1 per 160 children [3] but some epidemiological variations have been reported among world regions [3,4]. Possible explanations for this variability include dissimilarity in methods, variations in diagnostic or community identification, and potential risk factors [5]. To date, the Diagnostic and Statistical Manual of Mental Disorders (DSM-5), ASD comprises only two symptomatic domains: deficits in communication and social interaction, and repetitive and restricted behavior [6]. Thus, the current ASD definition includes autistic disorder, Asperger’s disorder, pervasive developmental disorder not otherwise specified (PDD-NOS), Rett’s disorder, and childhood disintegrative disorder, which represent a moderate variation in ASD diagnosis with respect to the previous DSM version [7]. Throughout life, the clinical course of ASD is variable and includes a wide range of clinical manifestations [8], including poor social skills, language, intellectual disabilities, sensory abnormalities (hyper- or hypo-sensory responsiveness), motor tics, and gross motor discoordination [8,9,10,11]. Nevertheless, ASD is a heterogeneous disorder with many inter-subject dissimilarities in social behaviors that some authors have associated with a combination of genetic variants that contribute to different phenotypic outcomes [12,13].

During the first years of life, the clinical symptoms correlate with two pathophysiological abnormalities in the brain. The overgrowth of certain brain regions precedes some symptoms of ASD and also coincides with periods of high neural plasticity [14,15]. From prenatal stages to birth, a slight delay in neural development is observed, which seems to be compensated by an accelerated brain growth during the first years of life [16,17]. This rapid overgrowth tends to reduce during the second and third years of life [14]. From the 5th to 16th year of life, this abnormal brain growth ceases and tends to normalize when compared to typically developing subjects [14,18]. In adults, some structures, such as the frontal lobe, cerebellum, and amygdala, show a normal or reduced volume as compared to typically developing subjects [19,20,21,22]. Recently, it has been reported that children with ASD show significant alterations in the connectivity between the right insula with the supramarginal gyrus and left superior frontal gyrus [23]. Adolescents with ASD also show age-dependent alterations in the connectivity between the frontal lobe and parietal region [24], which supports the notion that ASD is a dynamic developmental syndrome. Some of these anatomical abnormalities have been associated with extensive changes in myelination, excessive oxidative stress [25], glial activation [26], minicolumn pathology [27], abnormal neurogenesis, and neuronal migration [28,29]. Because the cerebral size in patients with ASD often correlates with functional deficits [30,31], these changes in the brain-growth pattern seem to be crucial to understand the symptomatology and etiology of ASD.

In the postnatal brain, white matter enlargement is one of the biological events that produces considerable changes in the volume of brain regions [32]. In the human brain, myelination is mostly a postnatal process that reaches its highest during childhood and continues until early adulthood [33]. Hence, myelination and oligodendrogenesis appear to be crucial events that notably modify several neuroanatomical structures. Increasing evidence suggests that this dynamic process may be disrupted in the ASD brain [34]. Patients with ASD show enlarged cerebellar and cerebral white matter structures and this increase occurs during the first years of life. This brain overgrowth of white matter observed in ASD is not sustained throughout life and tends to be smaller with age [14]. A pilot study in children diagnosed with autism between 4 and 6 years suggested that alterations in axons and myelin of the *corona radiata* appear to be associated with the clinical severity of ASD [35]. Additional evidence in adolescents indicates that ASD brains show fewer axons, less axonal volume, and a low density of white matter tracts in the corpus callosum, frontal-occipital fasciculus, right uncinate fasciculus, and right arcuate fasciculus [36]. Interestingly, pathological changes in the cytoarchitecture of white matter in all brain lobes [30] and altered myelination rate in corpus callosum [1,37] may explain the dysfunctional connectivity found in medial parietal and temporoparietal regions [16]. These structural disruptions have also been correlated to parental age [38] and may explain the severity of stereotypes and deficits in social interaction [31]. In contrast, clinical data indicate that the improvement in ASD symptoms is associated with white matter recovery [39]. In this review, we described the general pathogenesis and neuroanatomical changes found in several regions of the ASD brain and many of these areas correspond to white matter regions. Thus, we explained proteins, genes, and signaling pathways that regulate the oligodendroglial process, which in some cases are coincidentally affected in ASD. Finally, we compiled the most recent evidence that suggests a role for myelination and oligodendrocytes in the pathogenesis of ASD. Taken together, these reports suggest that some pathological changes observed in the white matter may explain the abnormal brain development related to ASD.

## 2. Pathophysiological Basis of ASD

ASD is considered by some authors as a brain connectivity disorder that, in turn, modifies the inhibition/excitation balance. Some reports sustain that the neuronal hypoconnectivity in frontal areas and fusiform face area observed in ASD patients [40] may explain the morphological alteration in brain size and interhemispheric connections [41,42,43]. This balance between neuronal inhibition and excitation is coordinated by several biological and molecular processes that modify synapse structure and brain plasticity [44], which may affect the frontotemporal, frontolimbic, frontoparietal, and interhemispheric connections [21]. Intriguingly, some local neuronal circuits in the frontal lobe are overconnected, whereas long-range connections (cortico-parietal, sub-cortical systems, and inter-hemispheric tracts) seem to be reduced in ASD patients [16,21,45,46].

However, abnormalities in neuronal connections per se cannot explain all the functional changes observed in ASD, and emerging evidence suggests that glial cells may play a pivotal role in neuroanatomical and behavioral changes found in ASD. Astrocytes and oligodendrocytes may contribute to neurochemical imbalances described in the autistic brain by disrupting neurotransmission or modifying axonal conduction. In mice models for ASD, the genetic depletion of glutamate transporter-1 (GLT-1) in astrocytes increases excitatory neurotransmission that is related to a high frequency of repetitive behaviors [44], whereas phosphatase and tensin homolog (PTEN) mislocation produces precocious maturation of oligodendrocytes that is associated with aberrant myelination [47]. A recent report suggests that a mutation in the eukaryotic translation initiation factor 4E (eIF4E) in microglia produces sex-dependent ASD-like behaviors by modifying synaptic development and function in male mice [48]. Therefore, neuroinflammation and pro-inflammatory cytokines released by microglia can alter gliotransmission, ion-channel expression, brain plasticity, and oxidative stress that may also lead to behavioral dysfunctions [49]. Interestingly, oxidative stress has also been implicated in the pathophysiological process of ASD by affecting the myelination process [50,51].

In this regard, oxidant radicals can damage the oligodendrocyte population that fails to differentiate into myelin-forming mature oligodendrocytes and increase a significant proliferation of oligodendrocyte precursor cells (OPCs) that, in turn, impair the whole myelination process [52]. Thus, oligodendrocytes are a very susceptible cell lineage to oxidant radicals because they have low levels of glutathione (a highly efficient antioxidant molecule) and elevated amounts of sphingolipids [53,54]. Therefore, oligodendrocytes represent a plausible cellular target for the oxidative stress identified in the ASD brain.

**Figure 1:**
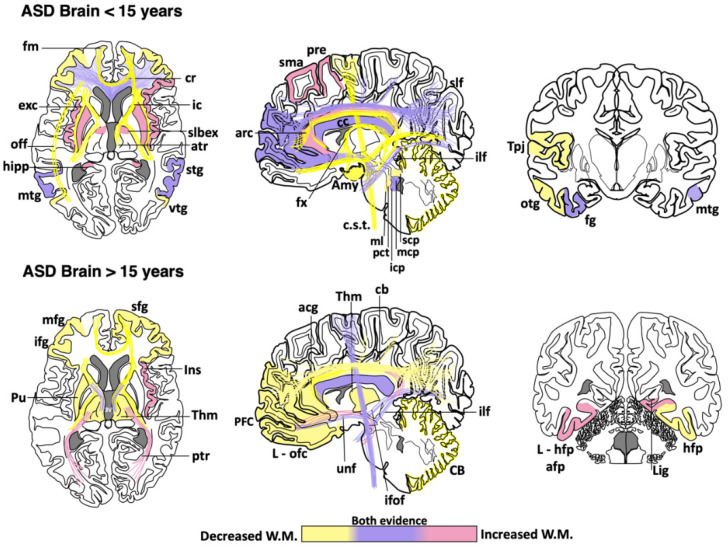
Cortical areas, subcortical areas and white matter tracts affected in patients with Autism Spectrum Disorder (ASD). White matter areas with the most noticeable changes are represented in the three anatomical planes of the human brain: axial, sagittal, and coronal, respectively. Brain schemes also indicate the most common myelination changes that occur in ASD patients during two different stages of development [21,30,36,60,64,65,67,70,71,72,73,74,75,76,77,78,79,80,81,82,83,84,85,86,87,88,89,90,91,92,93,94,95,96,97,98,99,100,101,102,103,104,105,106,107,108,109]. acg: anterior cingulate gyrus; afp: amygdala-fusiform pathway; Amy: amygdala; atr: anterior thalamic radiation; arc: arcuate fasciculus; CB: cerebellum; cb: cingulum bundle; cc: corpus callosum; c.s.t.: corticospinal tract; cr: corona radiata; exc: external capsule; fm: forceps minor; fx: fornix; fg: fusiform gyrus; hipp: hippocampus; icp: inferior cerebellar peduncle; ifof: inferior fronto-occipital fasciculus; ifg: inferior frontal gyrus; Ins: insula; ic: internal capsule; L-hfp: left hippocampus-fusiform pathway; L-ilf: Left inferior longitudinal fasciculus; L-ofc: left orbitofrontal cortex; lig: lingual gyrus; ml: medial lemniscus; mcp: middle cerebellar peduncle; mtg: middle frontal gyrus; mtg: middle temporal gyrus; off: occipitofrontal fasciculus; otg: occipitotemporal gyrus; pct: pontine crossing tracts; ptr: posterior thalamic radiation; pre: precentral area; PFC: prefrontal cortex; Pu: putamen; slbex: sub-lobar extranuclear area; scp: superior cerebellar peduncle; sfg: superior frontal gyrus; slf: superior longitudinal fasciculus; stg: superior temporal gyrus; sma: supplementary motor area; Tpj: temporoparietal junction; Thm: thalamus; unf: uncinate fasciculus; vtg: ventral temporal gyrus.

Abnormal development in the white matter and myelination has been found in animal models of ASD [55,56,57,58,59] and humans [60,61]. Neuroimaging studies support the notion that ASD patients have a complex and dynamic disorder [62] in which the level of white matter involvement is associated with clinical severity [60,63,64,65,66,67,68,69]. ASD patients show extensive alterations in the white matter of several cortical and subcortical regions, such as the orbitofrontal cortex [70], anterior cingulate cortex and lateral prefrontal cortex [61], external capsule [71,72], arcuate fasciculus [73,74,75], ventral temporal gyrus [60,76], temporo-parietal junctions [60,72,77], amygdala [60], occipitofrontal fasciculus [60], occipitotemporal gyrus [77], thalamus [78], superior and middle cerebellar peduncle [79,80], medial lemniscus [81], and the corticospinal tract [82,83,84] (Figure 1). However, some clinical reports have shown contradictory findings in the myelination process of ASD brains. From childhood to adolescence, the inferior longitudinal fasciculus shows pathological changes in the white matter density [83,84,85,86,87,88,89,90] (Table 1). The uncinate fasciculus of ASD patients younger than 15 years may show increased or decreased white matter density [64,65,74,75,91], but, after age 15, only an increase in the white matter has been found [87]. ASD patients older than 15 years may also have high white matter density in the lingual gyrus [78], amygdala-fusiform pathway, and left hippocampi-fusiform pathway [92].

Although a reduction in the white matter density and impaired structural integrity of corpus callosum appears to be a consistent finding throughout the clinical evolution of ASD [64,71,72,74,77,79,82,83,84,99,100,101,102], some clinical studies have found an increase in the genu and midbody subregion of the corpus callosum [94] or the entire corpus callosum [78]. This evidence indicates that white matter alterations might not be present in the whole white matter or restricted to certain portions of the corpus callosum [89,110,111]. The disparity in these clinical findings may be due to multiple factors, including age range, specific diagnosis, comorbidities, medication, gender, type of imaging, or sample size. Other factors such as inter-individual differences, nurture, and epigenetic factors can also explain the discrepancy among clinical studies. Nevertheless, these differences also suggest that ASD might be associated with abnormalities in the myelination process, which is a very dynamic event. Therefore, multicenter longitudinal studies are required to fully establish which brain regions are more susceptible to determine whether alterations in certain brain regions may be linked to some specific ASD phenotypes.

## 3. Role of Oligodendrocytes in ASD: Cellular and Molecular Evidence

**Figure 2:**
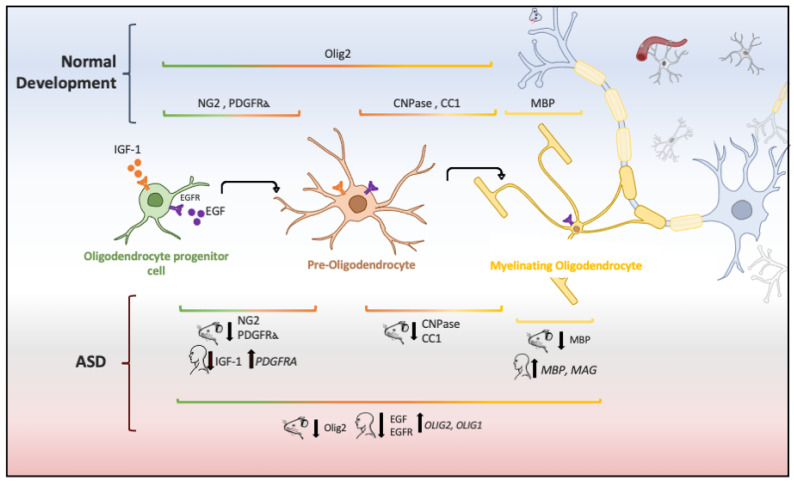
Oligodendrocyte lineage and molecular markers (genes or proteins) expressed under physiological and ASD conditions. Each human and mouse illustration indicates whether the molecular abnormality was found in clinical or experimental conditions [29,55,56,121,122,123,124].

In animal models of ASD, reduction in the proliferation of oligodendroglial cells and low levels of myelin basic protein (MBP) has also been implicated in ASD pathogenesis [55]. Oligodendrocytes are glial cells that myelinate the brain and spinal cord to insulate axons electrically and provide neurons with trophic and metabolic factors. Mature oligodendrocytes originate from OPCs that, throughout life, preserve the population of myelinating oligodendrocytes [112]. OPCs are a persistent cell population that consists of around 5% to 10% of the total number of cells in the adult brain [113,114]. White-matter regions contain a high number of oligodendrocytes and OPCs that are in contact with axons, which facilitate neuronal communication [115]. Migrating and resident OPCs express the plated-derived growth factor receptor alpha (PDGFRα) and the oligodendrocyte transcription factor 2 (Olig2), and NG2 proteoglycan (Figure 2), which are cell markers of these progenitor cells [29,116]. Remarkably, a subpopulation of NG2-expressing cells (NG2 glia) establish synaptic junctions with local neurons and modulates axonal conduction by releasing glutamate and stimulating α-Amino-3-hydroxy-5-methyl-4-isoxazole propionic acid (AMPA) receptors [117,118,119]. Experimental evidence has suggested that disruption in neuron–glia interactions promotes autistic-like features [120]. However, it is unknown whether NG2 glia or glial transmission is implicated directly in the pathophysiology or clinical manifestations of ASD patients.

The aberrant development of white matter found in patients with ASD (Figure 1) and disrupted protein levels in oligodendrocytes in ASD mice models (Figure 2) are suggestive of disruptions in the maturation of oligodendrocytes [40], which may convey changes in the white matter of corpus callosum and other white matter regions [21,36,40]. Deletions and duplications in the chromosome 15q11.2 region have been described in autistic patients and associated with alterations in myelination and abnormal development of the corpus callosum [125]. In humans, deletion carriers of 15q11.2 present a reduced volume of white matter [126]. This chromosomal region encodes CYFIP1 (Cytoplasmatic FMRP interacting protein 1), a protein that regulates cytoskeletal dynamics and protein translation. In mice, *Cyfip1* mutation reduces the myelin thickness and impairs neuronal connectivity in the corpus callosum that, in turn, produces aberrant behaviors and poor motor coordination in these mice [127]. Interestingly, deletions in chromosome 7 (7q11.23) have been associated with Williams Syndrome, a neurodevelopmental genetic disorder characterized by hypersociability and higher empathy [128,129], which has been also related to a decrease in the volume of white matter [130]. Therefore, disruptions in the myelination process could be associated with impairments in different chromosomes that, in turn, produce deficient electrical transmission resulting in impaired neurotransmitter release and behavioral manifestations [115].

Chromodomain helicase DNA binding proteins 7 (CHD-7) and 8 (CHD-8) are nucleosome remodeling factors that spatially and temporally control gene expression. CHD-7 is highly expressed in myelinating oligodendrocytes and its deficit or malfunction strongly compromises the myelination and remyelination process [131]. Transgenic *Chd7* mice demonstrate that this nucleosome remodeling factor is necessary throughout life for the transcription of *Sox10*, *Nkx2-2*, and *Gpr17* genes that regulate oligodendrocyte differentiation, and its inactivation decreases OPC survival and differentiation in cortex and corpus callosum via cellular tumor antigen p53 (Trp53) [131]. In contrast, CHD-8 is highly expressed in the early prenatal period and progressively decreases at later stages of development [132], but it remains highly expressed in the OPCs of adult white matter in the corpus callosum, optic nerve, and spinal cord [133]. CHD-8 regulates several genes associated with neurodevelopmental and synaptic functions that are affected in autism [134]. Interestingly, a clinical report found that *CHD8* mutations seem to be responsible for some phenotypic characteristics: macrocephaly and wide occipitofrontal circumference (head with a relatively large occipitofrontal diameter) and clinical behaviors (anxiety and social deficit) that have been associated with ASD [132,134]. A study in human neural stem cells and mid fetal human brain shows that the *CHD8* remodeling factor together with *ANK2*, *CUL3*, *DYRK1A*, *GRIN2B*, *KATNAL2*, *POGZ*, *SCN2A*, and *TBR1* are considered genetic risk factors to develop ASD [135]. Hence, this evidence suggests that *CHD7*/*CHD8* genes not only regulate the oligodendroglia differentiation and myelination but also provide some phenotypical features observed in people with ASD (macrocephaly, sleep dysfunction, growth retardation, and intellectual disability) [133,136].

Several growth factors regulate the proliferation and maturation of OPCs in the developing and adult brain. At the same time, systemic and local alterations in growth factors appear to be involved in the progression and severity of some psychiatric disorders [137]. Low levels of insulin-like growth factor 1 (IGF-1) reduce oligodendroglia survival [138] and cause OPCs to fail to differentiate into mature oligodendrocytes via bone morphogenetic protein (BMP) activation by up-regulating Noggin, Smad6, and Smad7 [139]. *Igf1* knockout mice show a reduction in the volume of the corpus callosum and anterior commissure that is associated with defectively myelinated axons and a decrease in the oligodendrocyte population [140]. Postnatally, these subjects also show fewer OPCs and mature oligodendrocytes that correlate with reduced expression of myelin proteins (MBP and the myelin proteolipid protein-PLP) [141]. Interestingly, children with ASD show low levels of IGF-1 in cerebrospinal fluid as compared with typically developing subjects [121,122]. In addition, low levels of IGF-1 are associated with deficient myelination and disorganization of neuronal circuits [142]. These pathological changes at the early stages of neurodevelopment may explain the impairment in synaptic development, inappropriate nerve conduction, and deficient axonal myelination frequently observed in ASD [142]. Therefore, IGF-1 and its signaling pathway are crucial for adequate axonal myelination and oligodendrocyte differentiation, but, when disrupted, white matter alterations, learning deficits, and ASD-like behaviors arise. To date, there are some experimental approaches to target the IGF-1 deficiency associated with ASD. The administration of IGF-1 for two weeks in Shank3-deficient mice, an ASD mouse model, notably improves the neuronal function by enhancing the long-term potentiation (LTP) and decreasing stereotypical behaviors [143]. Therefore, IGF-1 can be considered a promising therapeutic target, but further research is needed before using it in patients with ASD.

The Epidermal Growth Factor (EGF) and its receptors (ErbB 1–4) help preserve the oligodendrocyte population and repair demyelinating lesions by promoting proliferation, migration, and differentiation of OPCs in the adult brain [144,145,146,147]. Low levels of EGF and HER1 (ErbB1 in rodents) have been found in adults with high-functioning ASD [123]. In children, EGF levels correlate negatively with hyperactivity, tiptoe walking, and other motor signs [124]. Furthermore, the high expression of HER1 in children is correlated with high symptom severity (hyperactivity, conversational language, attention, eye contact, sound sensitivity, and expressive language) [148]. The interaction between neuregulin and ErbB4 protein, another member of the EGFR family, modifies the excitability of GABAergic neurons by increasing the inhibitory transmission [44,149]. GABAergic interneurons regulate cortical plasticity and cognitive flexibility in the frontal or parietal cortex [44]. Although the role of EGF in ASD has not been directly determined, this evidence suggests that changes in the ErbB family members or their ligands can modify the cortical plasticity and myelination as observed in ASD.

Both EGF and IGF-1 activate the PI3K/Akt/mTOR signaling pathway that regulates several intracellular functions, including cell growth, proliferation, differentiation, motility, survival, metabolism, and protein synthesis [150]. Akt/mTOR hyperactivity is commonly found in T cells [151] and peripheral blood [152] of patients with ASD. This hyperphosphorylation can, in turn, generate a deficiency of PTEN protein, a key negative regulator of the PI3K/Akt pathway [151], which results in Akt overactivation and increases the activity of mTOR [151,153]. The long-term activation of the Akt pathway prevents glutamate-mediated apoptosis in immature oligodendrocytes, which may induce aberrant myelination patterns [154]. Remarkably, patients with ASD and macrocephaly commonly show *PTEN* mutations [155]. In mice, disruption of PTEN activity by increasing Akt phosphorylation at Ser437 produces severe changes in the population of OPCs and in the genes and proteins involved in myelination (MBP, PLP, and myelin-associated glycoprotein-MAG), which induces social deficits that mimic some symptoms of ASD [156], such as increased anxiety and reduced social interest [153]. Interestingly, these animals also show aberrant myelin deposits adjacent to axons, increased volume of the corpus callosum, and brain enlargement [47,156,157]. Suppression of Akt/mTOR signaling improves ASD-associated symptoms in *Pten* knockout mice [153]. Therefore, *PTEN* alterations may be implicated in the pathogenesis of white matter abnormalities and behavioral symptoms observed in ASD [47,156,157]. A possible explanation for the low levels of IGF-1 and EGF reported in patients with ASD, despite the hyperactivity of the AKT/mTOR pathway, could be the interaction of receptors with other ligands as a compensatory mechanism, for instance, the IGF receptor (IGF1R) also interacts with insulin and insulin-like growth factor 2 (IGF-II) [158], whereas the EGFR has an affinity for other ligands such as TGF-ɑ and HB-EGF [159].

MAPK/ERK is another signaling pathway activated by IGF-1 and EGF [160] that regulates the differentiation, migration, proliferation, and survival of oligodendrocytes in the adult brain [161]. The MAPK/ERK signaling pathway also determines the morphology of oligodendrocytes [161] and regulates myelin synthesis [162] by promoting the activation of transcriptional factors (ELK1, AP2-complex, and CREB) that are necessary for the expression of MBP [162]. Interestingly, the crosstalk between PI3K/Akt/mTOR and MAPK pathways [163] controls the synthesis of oligodendroglia proteins [164] and OPCs proliferation [165]. However, while the role of MAPK/ERK in myelination and oligodendrocytes is well known, there is not enough evidence to clearly define the link between this signaling pathway and ASD. Hence, further research is required to elucidate this question.

## 4. Conclusions

ASD is a clinically heterogeneous disorder that seems to have a multifactorial origin. Typically, ASD has been considered as a brain connectivity disorder that produces a disparity in the inhibition/excitation balance. However, emerging evidence suggests that oligodendroglial cells play a role in the etiology of ASD. Oligodendrocytes insulate axons electrically and provide neurons with trophic factors that guarantee proper neurotransmission and axonal conduction in the white matter. Patients with ASD show an aberrant growth pattern in several white-matter regions that varies throughout life. During the first 15 years of life, axon tracts in the arcuate fasciculus, occipitofrontal fasciculus, and external capsule show a significant hypomyelination, whereas the fusiform gyrus and hippocampal area show a substantial increase in their myelin volume (Figure 1). In both cases, these alterations tend to normalize throughout development. Intriguingly, other brain regions such as cerebellum, cingulum, and internal capsule remain hypomyelinated. This atypical myelination pattern has been linked to several factors, including gene disruption and epigenetic factors. Some genes and growth factors implicated in this abnormal myelination process include Olig1, Olig2, *Sox10*, *PGFRA*, *Nkx2-2*, *Gpr17*, IGF-1, and EGF. Hence, this emerging evidence supports the notion that oligodendrogenesis and neural myelination may play a pivotal role in the pathophysiology of ASD and its clinical presentation. Understanding the molecular mediators involved in abnormal myelination patterns in the ASD brain may represent an initial step to design therapeutic targets that help improve social skills and disruptive behaviors observed in these patients.

## Figures and Tables

**Table 1:** Main changes observed in white matter regions of the ASD human brain. Age categorization was used to highlight the findings described in children and early adolescence (<15 years) vs. those observed in late adolescence and adults (>15 years). Asterisks indicate the brain regions where contradictory findings have been reported [21,30,36,60,64,65,67,70,71,72,73,74,75,76,77,78,79,80,81,82,83,84,85,86,87,88,89,90,91,92,93,94,95,96,97,98,99,100,101,102,103,104,105,106,107,108,109].

Age	Affected Brain Regions in ASD
<15 years	Increased white matter density: Supplementary motor area, left precentral, superior longitudinal fasciculus *, left cingulum *, right cingulate gyrus, prefrontal cortex *, radiate volume, corpus callosum *, right inferior frontal gyrus, putamen, insula, sublobar extranuclear area, right superior temporal gyrus, hippocampus, middle temporal gyrus *, fusiform gyrus, uncinate fasciculus *, inferior longitudinal fasciculus *, bilateral middle *, and left inferior cerebellar peduncle. Reduced white matter density: Superior longitudinal fasciculus *, cingulum *, cingulate gyrus, prefrontal cortex *, corona radiata, middle frontal gyrus, corpus callosum *, arcuate fasciculus, inferior frontal gyrus, forceps minor, fornix, anterior thalamic radiation, internal and external capsule, superior temporal gyrus, superior temporal sulcus, temporoparietal junctions, middle temporal gyrus *, right inferior frontal gyrus-middle temporal gyrus tracts, bilateral inferior frontal gyrus-fusiform gyrus tracts, inferior fronto-occipital fasciculus, occipitotemporal gyrus, uncinate fasciculus *, inferior longitudinal fasciculus *, amygdala, inferior temporal gyrus, bilateral superior, middle * and right inferior cerebellar peduncle, pontine crossing tracts and medial lemniscus, cerebellum, and corticospinal tract.
>15 years	Increased white matter density: Corpus callosum *, anterior and posterior thalamic radiation, right insula, bilateral amygdala-fusiform pathways temporal, left hippocampus-fusiform pathways, temporal segment of Superior longitudinal fasciculus *, right lingual gyrus, uncinate fasciculus, inferior fronto-occipital fasciculus *, inferior longitudinal fasciculus, and corticospinal tract *. Reduced white matter density: Superior longitudinal fasciculus, intraparietal sulcus, cingulum, anterior cingulate gyrus, right superior frontal gyrus, prefrontal cortex, middle frontal gyrus, corpus callosum *, left orbitofrontal cortex, inferior frontal gyrus, left putamen tracts, thalamus, forceps minor, anterior thalamic radiation, internal capsule, right hippocampus-fusiform pathway, inferior fronto-occipital fasciculus *, cerebellum, and corticospinal tract *.

## References

[1] Parellada M., Penzol M.J., Pina L., Moreno C., González-Vioque E., Zalsman G., Arango C. (2014). The neurobiology of autism spectrum disorders. Eur. Psychiatry.

[2] Carlisi C.O., Norman L.J., Lukito S.S., Radua J., Mataix-Cols D., Rubia K. (2017). Comparative multimodal meta-analysis of structural and functional brain abnormalities in autism spectrum disorder and obsessive-compulsive disorder. Biol. Psychiatry.

[3] Elsabbagh M., Divan G., Koh Y.J., Kim Y.S., Kauchali S., Marcín C., Montiel-Nava C., Patel V., Paula C.S., Wang C. (2012). Global prevalence of autism and other pervasive developmental disorders. Autism Res..

[4] Baxter A.J., Brugha T.S., Erskine H.E., Scheurer R.W., Vos T., Scott J.G. (2015). The epidemiology and global burden of autism spectrum disorders. Psychol. Med..

[5] Rice C.E., Rosanoff M., Dawson G., Durkin M.S., Croen L.A., Singer A., Yeargin-Allsopp M. (2012). Evaluating changes in the prevalence of the autism spectrum disorders (ASDs). Public Health Rev..

[6] American Psychiatric Association (2013). Diagnostic and Statistical Manual of Mental Disorders (DSM-5).

[7] Harstad E.B., Fogler J., Sideridis G., Weas S., Mauras C., Barbaresi W.J. (2015). Comparing diagnostic outcomes of autism spectrum disorder using DSM-IV-TR and DSM-5 criteria. J. Autism Dev. Disord..

[8] Masi A., DeMayo M.M., Glozier N., Guastella A.J. (2017). An overview of autism spectrum disorder, heterogeneity and treatment options. Neurosci. Bull..

[9] Balasco L., Provenzano G., Bozzi Y. (2020). Sensory abnormalities in autism spectrum disorders: A focus on the tactile domain, from genetic mouse models to the clinic. Front. Psychiatry.

[10] Mosconi M.W., Sweeney J.A. (2015). Sensorimotor dysfunctions as primary features of autism spectrum disorders. Sci. China Life Sci..

[11] Baum S.H., Stevenson R.A., Wallace M.T. (2015). Behavioral, perceptual, and neural alterations in sensory and multisensory function in autism spectrum disorder. Prog. Neurobiol..

[12] Girirajan S., Rosenfeld J.A., Coe B.P., Parikh S., Friedman N., Goldstein A., Filipink R.A., McConnell J.S., Angle B., Meschino W.S. (2012). Phenotypic heterogeneity of genomic disorders and rare copy-number variants. N. Engl. J. Med..

[13] Rylaarsdam L., Guemez-Gamboa A. (2019). Genetic causes and modifiers of autism spectrum disorder. Front. Cell. Neurosci..

[14] Courchesne E., Karns C.M., Davis H.R., Ziccardi R., Carper R.A., Tigue Z.D., Chisum H.J., Moses P., Pierce K., Lord C. (2001). Unusual brain growth patterns in early life in patients with autistic disorder: An MRI study. Neurology.

[15] Courchesne E., Carper R., Akshoomoff N. (2003). Evidence of brain overgrowth in the first year of life in autism. J. Am. Med. Assoc..

[16] Courchesne E., Pierce K. (2005). Why the frontal cortex in autism might be talking only to itself: Local over-connectivity but long-distance disconnection. Curr. Opin. Neurobiol..

[17] Courchesne E., Pierce K., Schumann C.M., Redcay E., Buckwalter J.A., Kennedy D.P., Morgan J. (2007). Mapping early brain development in autism. Neuron.

[18] Courchesne E., Campbell K., Solso S. (2011). Brain growth across the life span in autism: Age-specific changes in anatomical pathology. Brain Res..

[19] Morgan J.T., Barger N., Amaral D.G., Schumann C.M. (2014). Stereological study of amygdala glial populations in adolescents and adults with autism spectrum disorder. PLoS ONE.

[20] McAlonan G.M., Daly E., Kumari V., Critchley H.D., Van Amelsvoort T., Suckling J., Simmons A., Sigmundsson T., Greenwood K., Russell A. (2002). Brain anatomy and sensorimotor gating in Asperger’s syndrome. Brain.

[21] Bloemen O.J.N., Deeley Q., Sundram F., Daly E.M., Barker G.J., Jones D.K., Van Amelsvoort T.A.M.J., Schmitz N., Robertson D., Murphy K.C. (2010). White matter integrity in Asperger syndrome: A preliminary diffusion tensor magnetic resonance imaging study in adults. Autism Res..

[22] Schmitz N., Daly E., Murphy D. (2007). Frontal anatomy and reaction time in Autism. Neurosci. Lett..

[23] Rashid B., Blanken L.M.E., Muetzel R.L., Miller R., Damaraju E., Arbabshirani M.R., Erhardt E.B., Verhulst F.C., van der Lugt A., Jaddoe V.W.V. (2018). Connectivity dynamics in typical development and its relationship to autistic traits and autism spectrum disorder. Hum. Brain Mapp..

[24] Lawrence K.E., Hernandez L.M., Bookheimer S.Y., Dapretto M. (2019). Atypical longitudinal development of functional connectivity in adolescents with autism spectrum disorder. Autism Res..

[25] Chauhan A., Chauhan V. (2006). Oxidative stress in autism. Pathophysiology.

[26] Zeidán-Chuliá F., Salmina A.B., Malinovskaya N.A., Noda M., Verkhratsky A., Moreira J.C.F. (2014). The glial perspective of autism spectrum disorders. Neurosci. Biobehav. Rev..

[27] Casanova M.F., Buxhoeveden D.P., Switala A.E., Roy E. (2002). Minicolumnar pathology in autism. Neurology.

[28] Wegiel J., Kuchna I., Nowicki K., Imaki H., Wegiel J., Marchi E., Ma S.Y., Chauhan A., Chauhan V., Bobrowicz T.W. (2010). The neuropathology of autism: Defects of neurogenesis and neuronal migration, and dysplastic changes. Acta Neuropathol..

[29] Zeidán-Chuliá F., de Oliveira B.H.N., Casanova M.F., Casanova E.L., Noda M., Salmina A.B., Verkhratsky A. (2016). Up-regulation of oligodendrocyte lineage markers in the cerebellum of autistic patients: Evidence from network analysis of gene expression. Mol. Neurobiol..

[30] Herbert M.R., Ziegler D.A., Makris N., Filipek P.A., Kemper T.L., Normandin J.J., Sanders H.A., Kennedy D.N., Caviness V.S. (2004). Localization of white matter volume increase in autism and developmental language disorder. Ann. Neurol..

[31] Hong S.J., Hyung B., Paquola C., Bernhardt B.C. (2019). The superficial white matter in autism and its role in connectivity anomalies and symptom severity. Cereb. Cortex.

[32] Mengler L., Khmelinskii A., Diedenhofen M., Po C., Staring M., Lelieveldt B.P.F., Hoehn M. (2014). Brain maturation of the adolescent rat cortex and striatum: Changes in volume and myelination. Neuroimage.

[33] Tomassy G.S., Dershowitz L.B., Arlotta P. (2016). Diversity matters: A revised guide to myelination. Trends Cell Biol..

[34] Herbert M.R., Ziegler D.A., Deutsch C.K., O’Brien L.M., Lange N., Bakardjiev A., Hodgson J., Adrien K.T., Steele S., Makris N. (2003). Dissociations of cerebral cortex, subcortical and cerebral white matter volumes in autistic boys. Brain.

[35] Carmody D.P., Lewis M. (2010). Regional white matter development in children with autism spectrum disorders. Dev. Psychobiol..

[36] Dimond D., Schuetze M., Smith R.E., Dhollander T., Cho I., Vinette S., Ten Eycke K., Lebel C., McCrimmon A., Dewey D. (2019). Reduced white matter fiber density in autism spectrum disorder. Cereb. Cortex.

[37] DiCicco-Bloom E., Lord C., Zwaigenbaum L., Courchesne E., Dager S.R., Schmitz C., Schultz R.T., Crawley J., Young L.J. (2006). The developmental neurobiology of autism spectrum disorder. J. Neurosci..

[38] Yassin W., Kojima M., Owada K., Kuwabara H., Gonoi W., Aoki Y., Takao H., Natsubori T., Iwashiro N., Kasai K. (2019). Paternal age contribution to brain white matter aberrations in autism spectrum disorder. Psychiatry Clin. Neurosci..

[39] Swanson M.R., Hazlett H.C. (2019). White matter as a monitoring biomarker for neurodevelopmental disorder intervention studies. J. Neurodev. Disord..

[40] Hughes J.R. (2007). Autism: The first firm finding = underconnectivity?. Epilepsy Behav..

[41] Boger-Megiddo I., Shaw D.W.W., Friedman S.D., Sparks B.F., Artru A.A., Giedd J.N., Dawson G., Dager S.R. (2006). Corpus callosum morphometrics in young children with autism spectrum disorder. J. Autism Dev. Disord..

[42] Dawson G., Munson J., Webb S.J., Nalty T., Abbott R., Toth K. (2007). Rate of head growth decelerates and symptoms worsen in the second year of life in autism. Biol Psychiatry.

[43] Wolff J.J., Gu H., Gerig G., Elison J.T., Styner M., Gouttard S., Botteron K.N., Dager S.R., Dawson G., Estes A.M. (2012). Differences in white matter fiber tract development present from 6 to 24 months in infants with autism. Am. J. Psychiatry.

[44] Lee E., Lee J., Kim E. (2017). Excitation/inhibition imbalance in animal models of autism spectrum disorders. Biol. Psychiatry.

[45] Ecker C., Ronan L., Feng Y., Daly E., Murphy C., Ginestet C.E., Brammer M., Fletcher P.C., Bullmore E.T., Suckling J. (2013). Intrinsic gray-matter connectivity of the brain in adults with autism spectrum disorder. Proc. Natl. Acad. Sci. USA.

[46] O’Reilly C., Lewis J.D., Elsabbagh M. (2017). Is functional brain connectivity atypical in autism? A systematic review of EEG and MEG studies. PLoS ONE.

[47] Lee H., Thacker S., Sarn N., Dutta R., Eng C. (2019). Constitutional mislocalization of Pten drives precocious maturation in oligodendrocytes and aberrant myelination in model of autism spectrum disorder. Transl. Psychiatry.

[48] Xu Z.X., Kim G.H., Tan J.W., Riso A.E., Sun Y., Xu E.Y., Liao G.Y., Xu H., Lee S.H., Do N.Y. (2020). Elevated protein synthesis in microglia causes autism-like synaptic and behavioral aberrations. Nat. Commun..

[49] Kim Y.S., Choi J., Yoon B.E. (2020). Neuron-glia interactions in neurodevelopmental disorders. Cells.

[50] Chauhan A., Chauhan W., Brown W.T., Evans T.A., Perry G., Smith M.A., Salomon R.G., McGinnis W.R., Sajdel-Sulkowska M., Zhu X. (2010). Autism oxidative stress, inflamation and Immune abnormalities. CRC Press.

[51] Yui K., Kawasaki Y., Yamada H., Ogawa S. (2016). Oxidative stress and nitric oxide in autism spectrum disorder and other neuropsychiatric disorders. CNS Neurol. Disord. Drug Targets.

[52] Back S.A. (2017). White matter injury in the preterm infant: Pathology and mechanisms. Acta Neuropathol..

[53] Thorburne S.K., Juurlink B.H.J. (1996). Low glutathione and high iron govern the susceptibility of oligodendroglial precursors to oxidative stress. J. Neurochem..

[54] McTigue D.M., Tripathi R.B. (2008). The life, death, and replacement of oligodendrocytes in the adult CNS. J. Neurochem..

[55] Graciarena M., Seiffe A., Nait-Oumesmar B., Depino A.M. (2019). Hypomyelination and oligodendroglial alterations in a mouse model of autism spectrum disorder. Front. Cell. Neurosci..

[56] Khanbabaei M., Hughes E., Ellegood J., Qiu L.R., Yip R., Dobry J., Murari K., Lerch J.P., Rho J.M., Cheng N. (2019). Precocious myelination in a mouse model of autism. Transl. Psychiatry.

[57] Cartocci V., Catallo M., Tempestilli M., Segatto M., Pfrieger F.W., Bronzuoli M.R., Scuderi C., Servadio M., Trezza V., Pallottini V. (2018). Altered brain cholesterol/isoprenoid metabolism in a rat model of autism spectrum disorders. Neuroscience.

[58] Pacey L.K.K., Xuan I.C.Y., Guan S., Sussman D., Henkelman R.M., Chen Y., Thomsen C., Hampson D.R. (2013). Delayed myelination in a mouse model of fragile X syndrome. Hum. Mol. Genet..

[59] Meikle L., Talos D.M., Onda H., Pollizzi K., Rotenberg A., Sahin M., Jensen F.E., Kwiatkowski D.J. (2007). A mouse model of tuberous sclerosis: Neuronal loss of Tsc1 causes dysplastic and ectopic neurons, reduced myelination, seizure activity, and limited survival. J. Neurosci..

[60] Noriuchi M., Kikuchi Y., Yoshiura T., Kira R., Shigeto H., Hara T., Tobimatsu S., Kamio Y. (2010). Altered white matter fractional anisotropy and social impairment in children with autism spectrum disorder. Brain Res..

[61] Zikopoulos B., García-Cabezas M.Á., Barbas H. (2018). Parallel trends in cortical gray and white matter architecture and connections in primates allow fine study of pathways in humans and reveal network disruptions in autism. PLoS Biol..

[62] Ha S., Sohn I., Kim N., Sim H.J., Cheon K. (2015). Characteristics of brains in autism spectrum disorder: Structure, function and connectivity across the lifespan. Exp. Neurobiol..

[63] Wolff J.J., Gerig G., Lewis J.D., Soda T., Styner M.A., Vachet C., Botteron K.N., Elison J.T., Dager S.R., Estes A.M. (2015). Altered corpus callosum morphology associated with autism over the first 2 years of life. Brain.

[64] Cheon K.A., Kim Y.S., Oh S.H., Park S.Y., Yoon H.W., Herrington J., Nair A., Koh Y.J., Jang D.P., Kim Y.B. (2011). Involvement of the anterior thalamic radiation in boys with high functioning autism spectrum disorders: A diffusion tensor imaging study. Brain Res..

[65] Kumar A., Sundaram S.K., Sivaswamy L., Behen M.E., Makki M.I., Ager J., Janisse J., Chugani H.T., Chugani D.C. (2010). Alterations in frontal lobe tracts and corpus callosum in young children with autism spectrum disorder. Cereb. Cortex.

[66] Schaer M., Ottet M.-C., Scariati E., Dukes D., Franchini M., Eliez S., Glaser B. (2013). Decreased frontal gyrification correlates with altered connectivity in children with autism. Front. Hum. Neurosci..

[67] Ikuta T., Shafritz K.M., Bregman J., Peters B.D., Gruner P., Malhotra A.K., Szeszko P.R. (2014). Abnormal cingulum bundle development in autism: A probabilistic tractography study. Psychiatry Res. Neuroimaging.

[68] Nair A., Treiber J.M., Shukla D.K., Shih P., Müller R.A. (2013). Impaired thalamocortical connectivity in autism spectrum disorder: A study of functional and anatomical connectivity. Brain.

[69] Pardini M., Elia M., Garaci F.G., Guida S., Coniglione F., Krueger F., Benassi F., Emberti Gialloreti L. (2012). Long-term cognitive and behavioral therapies, combined with augmentative communication, are related to uncinate fasciculus integrity in autism. J. Autism Dev. Disord..

[70] Pardini M., Garaci F.G., Bonzano L., Roccatagliata L., Palmieri M.G., Pompili E., Coniglione F., Krueger F., Ludovici A., Floris R. (2009). White matter reduced streamline coherence in young men with autism and mental retardation. Eur. J. Neurol..

[71] McAlonan G.M., Cheung C., Cheung V., Wong N., Suckling J., Chua S.E. (2009). Differential effects on white-matter systems in high-functioning autism and Asperger’s syndrome. Psychol. Med..

[72] Barnea-Goraly N., Lotspeich L.J., Reiss A.L. (2010). Similar white matter aberrations in children with autism and their unaffected siblings. Arch. Gen. Psychiatry.

[73] Fletcher P.T., Whitaker R.T., Tao R., DuBray M.B., Froehlich A., Ravichandran C., Alexander A.L., Bigler E.D., Lange N., Lainhart J.E. (2010). Microstructural connectivity of the arcuate fasciculus in adolescents with high-functioning autism. Neuroimage.

[74] Jeong J.W., Kumar A.K., Sundaram S.K., Chugani H.T., Chugani D.C. (2011). Sharp curvature of frontal lobe white matter pathways in children with autism spectrum disorders: Tract-based morphometry analysis. Am. J. Neuroradiol..

[75] Lo Y.C., Soong W.T., Gau S.S.F., Wu Y.Y., Lai M.C., Yeh F.C., Chiang W.Y., Kuo L.W., Jaw F.S., Tseng W.Y.I. (2011). The loss of asymmetry and reduced interhemispheric connectivity in adolescents with autism: A study using diffusion spectrum imaging tractography. Psychiatry Res. Neuroimaging.

[76] Cheung C., Chua S.E., Cheung V., Khong P.L., Tai K.S., Wong T.K.W., Ho T.P., McAlonan G.M. (2009). White matter fractional anisotrophy differences and correlates of diagnostic symptoms in autism. J. Child Psychol. Psychiatry Allied Discip..

[77] Barnea-Goraly N., Kwon H., Menon V., Eliez S., Lotspeich L., Reiss A.L. (2004). White matter structure in autism: Preliminary evidence from diffusion tensor imaging. Biol. Psychiatry.

[78] Ecker C., Rocha-Rego V., Johnston P., Mourao-Miranda J., Marquand A., Daly E.M., Brammer M.J., Murphy C., Murphy D.G. (2010). Investigating the predictive value of whole-brain structural MR scans in autism: A pattern classification approach. Neuroimage.

[79] Shukla D.K., Keehn B., Lincoln A.J., Müller R.A. (2010). White matter compromise of callosal and subcortical fiber tracts in children with autism spectrum disorder: A diffusion tensor imaging study. J. Am. Acad. Child Adolesc. Psychiatry.

[80] Sivaswamy L., Kumar A., Rajan D., Behen M., Muzik O., Chugani D., Chugani H. (2010). A diffusion tensor imaging study of the cerebellar pathways in children with autism spectrum disorder. J. Child Neurol..

[81] Yu Q., Peng Y., Kang H., Peng Q., Ouyang M., Slinger M., Hu D., Shou H., Fang F., Huang H. (2020). Differential white matter maturation from birth to 8 years of age. Cereb. Cortex.

[82] Brito A.R., Vasconcelos M.M., Domingues R.C., Hygino Da Cruz L.C., Rodrigues L.D.S., Gasparetto E.L., Calçada C.A.B.P. (2009). Diffusion tensor imaging findings in school-aged autistic children. J. Neuroimaging.

[83] Jou R.J., Mateljevic N., Kaiser M.D., Sugrue D.R., Volkmar F.R., Pelphrey K.A. (2011). Structural neural phenotype of autism: Preliminary evidence from a diffusion tensor imaging study using tract-based spatial statistics. Am. J. Neuroradiol..

[84] Shukla D.K., Keehn B., Müller R.A. (2011). Tract-specific analyses of diffusion tensor imaging show widespread white matter compromise in autism spectrum disorder. J. Child Psychol. Psychiatry Allied Discip..

[85] Sahyoun C.P., Belliveau J.W., Mody M. (2010). White matter integrity and pictorial reasoning in high-functioning children with autism. Brain Cogn..

[86] Ameis S.H., Fan J., Rockel C., Voineskos A.N., Lobaugh N.J., Soorya L., Wang A.T., Hollander E., Anagnostou E. (2011). Impaired structural connectivity of socio-emotional circuits in autism spectrum disorders: A diffusion tensor imaging study. PLoS ONE.

[87] Thomas C., Humphreys K., Jung K.J., Minshew N., Behrmann M. (2011). The anatomy of the callosal and visual-association pathways in high-functioning autism: A DTI tractography study. Cortex.

[88] Itahashi T., Yamada T., Nakamura M., Watanabe H., Yamagata B., Jimbo D., Shioda S., Kuroda M., Toriizuka K., Kato N. (2015). Linked alterations in gray and white matter morphology in adults with high-functioning autism spectrum disorder: A multimodal brain imaging study. Neuroimage Clin..

[89] Roine U., Salmi J., Roine T., Wendt T.N.-v., Leppämäki S., Rintahaka P., Tani P., Leemans A., Sams M. (2015). Constrained spherical deconvolution-based tractography and tract-based spatial statistics show abnormal microstructural organization in Asperger syndrome. Mol. Autism.

[90] Karahanoğlu F.I., Baran B., Nguyen Q.T.H., Meskaldji D.E., Yendiki A., Vangel M., Santangelo S.L., Manoach D.S. (2018). Diffusion-weighted imaging evidence of altered white matter development from late childhood to early adulthood in autism spectrum disorder. Neuroimage Clin..

[91] Poustka L., Jennen-Steinmetz C., Henze R., Vomstein K., Haffner J., Sieltjes B. (2012). Fronto-temporal disconnectivity and symptom severity in children with autism spectrum disorder. World J. Biol. Psychiatry.

[92] Conturo T.E., Williams D.L., Smith C.D., Gultepe E., Akbudak E., Minshew N.J. (2008). Neuronal fiber pathway abnormalities in autism: An initial MRI diffusion tensor tracking study of hippocampo-fusiform and amygdalo-fusiform pathways. J. Int. Neuropsychol. Soc..

[93] Cheng Y., Chou K.H., Chen I.Y., Fan Y.T., Decety J., Lin C.P. (2010). Atypical development of white matter microstructure in adolescents with autism spectrum disorders. Neuroimage.

[94] Weinstein M., Ben-Sira L., Levy Y., Zachor D.A., Itzhak E.B.e.n., Artzi M., Tarrasch R., Eksteine P.M., Hendler T., Bashat D. (2011). Ben Abnormal white matter integrity in young children with autism. Hum. Brain Mapp..

[95] Mengotti P., D’Agostini S., Terlevic R., De Colle C., Biasizzo E., Londero D., Ferro A., Rambaldelli G., Balestrieri M., Zanini S. (2011). Altered white matter integrity and development in children with autism: A combined voxel-based morphometry and diffusion imaging study. Brain Res. Bull..

[96] Ke X., Tang T., Hong S., Hang Y., Zou B., Li H., Zhou Z., Ruan Z., Lu Z., Tao G. (2009). White matter impairments in autism, evidence from voxel-based morphometry and diffusion tensor imaging. Brain Res..

[97] Li Y., Zhou Z., Chang C., Qian L., Li C., Xiao T., Xiao X., Chu K., Fang H., Ke X. (2019). Anomalies in uncinate fasciculus development and social defects in preschoolers with autism spectrum disorder. BMC Psychiatry.

[98] Sahyoun C.P., Belliveau J.W., Soulières I., Schwartz S., Mody M. (2010). Neuroimaging of the functional and structural networks underlying visuospatial vs. linguistic reasoning in high-functioning autism. Neuropsychologia.

[99] Vidal C.N., Nicolson R., DeVito T.J., Hayashi K.M., Geaga J.A., Drost D.J., Williamson P.C., Rajakumar N., Sui Y., Dutton R.A. (2006). Mapping corpus callosum deficits in autism: An index of aberrant cortical connectivity. Biol. Psychiatry.

[100] Frazier T.W., Keshavan M.S., Minshew N.J., Hardan A.Y. (2012). A two-year longitudinal MRI study of the corpus callosum in autism. J. Autism Dev. Disord..

[101] Hardan A.Y., Pabalan M., Gupta N., Bansal R., Melhem N.M., Fedorov S., Keshavan M.S., Minshew N.J. (2009). Corpus callosum volume in children with autism. Psychiatry Res. Neuroimaging.

[102] Hong S., Ke X., Tang T., Hang Y., Chu K., Huang H., Ruan Z., Lu Z., Tao G., Liu Y. (2011). Detecting abnormalities of corpus callosum connectivity in autism using magnetic resonance imaging and diffusion tensor tractography. Psychiatry Res. Neuroimaging.

[103] Jou R.J., Jackowski A.P., Papademetris X., Rajeevan N., Staib L.H., Volkmar F.R. (2011). Diffusion tensor imaging in autism spectrum disorders: Preliminary evidence of abnormal neural connectivity. Aust. N. Z. J. Psychiatry.

[104] Thakkar K.N., Polli F.E., Joseph R.M., Tuch D.S., Hadjikhani N., Barton J.J.S., Manoach D.S. (2008). Response monitoring, repetitive behaviour and anterior cingulate abnormalities in autism spectrum disorders (ASD). Brain.

[105] Haigh S.M., Keller T.A., Minshew N.J., Eack S.M. (2020). Reduced white matter integrity and deficits in neuropsychological functioning in adults with autism spectrum disorder. Autism Res..

[106] Catani M., Jones D.K., Daly E., Embiricos N., Deeley Q., Pugliese L., Curran S., Robertson D., Murphy D.G.M. (2008). Altered cerebellar feedback projections in Asperger syndrome. Neuroimage.

[107] Hardan A.Y., Minshew N.J., Keshavan M.S. (2000). Corpus callosum size in autism. Neurology.

[108] Piven J., Bailey J., Ranson B.J., Arndt S. (1997). An MRI study of the corpus callosum in autism. Am. J. Psychiatry.

[109] Langen M., Leemans A., Johnston P., Ecker C., Daly E., Murphy C.M., Dell’Acqua F., Durston S., Murphy D.G.M. (2012). Fronto-striatal circuitry and inhibitory control in autism: Findings from diffusion tensor imaging tractography. Cortex.

[110] Libero L.E., DeRamus T.P., Lahti A.C., Deshpande G., Kana R.K. (2015). Multimodal neuroimaging based classification of autism spectrum disorder using anatomical, neurochemical, and white matter correlates. Cortex.

[111] Haigh S.M., Eack S.M., Keller T., Minshew N.J., Behrmann M. (2019). White matter structure in schizophrenia and autism: Abnormal diffusion across the brain in schizophrenia. Neuropsychologia.

[112] Bradl M., Lassmann H. (2010). Oligodendrocytes: Biology and pathology. Acta Neuropathol..

[113] Dulamea A. (2017). The contribution of oligodendrocytes and oligodendrocyte progenitor cells to central nervous system repair in multiple sclerosis: Perspectives for remyelination therapeutic strategies. Neural Regen. Res..

[114] Tiane A., Schepers M., Rombaut B., Hupperts R., Prickaerts J., Hellings N., van den Hove D., Vanmierlo T. (2019). From OPC to oligodendrocyte: An Epigenetic journey. Cells.

[115] Alexander A.L., Hurley S.A., Samsonov A.A., Adluru N., Hosseinbor A.P., Mossahebi P., Tromp D.P.M., Zakszewski E., Field A.S. (2011). Characterization of cerebral white matter properties using quantitative magnetic resonance imaging stains. Brain Connect..

[116] Menn B., Garcia-Verdugo J.M., Yaschine C., Gonzalez-Perez O., Rowitch D., Alvarez-Buylla A. (2006). Origin of oligodendrocytes in the subventricular zone of the adult brain. J. Neurosci..

[117] Bergles D.E., Roberts J.D.B., Somogyl P., Jahr C.E. (2000). Glutamatergic synapses on oligodendrocyte precursor cells in the hippocampus. Nature.

[118] Kukley M., Capetillo-Zarate E., Dietrich D. (2007). Vesicular glutamate release from axons in white matter. Nat. Neurosci..

[119] Ziskin J.L., Nishiyama A., Rubio M., Fukaya M., Bergles D.E. (2007). Vesicular release of glutamate from unmyelinated axons in white matter. Nat. Neurosci..

[120] Bronzuoli M.R., Facchinetti R., Ingrassia D., Sarvadio M., Schiavi S., Steardo L., Verkhratsky A., Trezza V., Scuderi C. (2018). Neuroglia in the autistic brain: Evidence from a preclinical model 11 medical and health sciences 1109 neurosciences 17 psychology and cognitive sciences 1701 psychology. Mol. Autism.

[121] Riikonen R., Makkonen I., Vanhala R., Turpeinen U., Kuikka J., Kokki H. (2006). Cerebrospinal fluid insulin-like growth factors IGF-1 and IGF-2 in infantile autism. Dev. Med. Child Neurol..

[122] Vanhala R., Turpeinen U., Riikonen R. (2007). Low levels of insulin-like growth factor-I in cerebrospinal fluid in children with autism. Dev. Med. Child Neurol..

[123] Suzuki K., Hashimoto K., Iwata Y., Nakamura K., Tsujii M., Tsuchiya K.J., Sekine Y., Suda S., Sugihara G., Matsuzaki H. (2007). Decreased serum levels of epidermal growth factor in adult subjects with high-functioning autism. Biol. Psychiatry.

[124] Russo A.J. (2013). Decreased epidermal growth factor (EGF) associated with HMGB1 and increased hyperactivity in children with autism. Biomark. Insights.

[125] Silva A.I., Ulfarsson M.O., Stefansson H., Gustafsson O., Walters G.B., Linden D.E.J., Wilkinson L.S., Drakesmith M., Owen M.J., Hall J. (2019). Reciprocal white matter changes associated with copy number variation at 15q11.2 BP1-BP2: A diffusion tensor imaging study. Biol. Psychiatry.

[126] Stefansson H., Meyer-Lindenberg A., Steinberg S., Magnusdottir B., Morgen K., Arnarsdottir S., Bjornsdottir G., Walters G.B., Jonsdottir G.A., Doyle O.M. (2014). CNVs conferring risk of autism or schizophrenia affect cognition in controls. Nature.

[127] Domínguez-Iturza N., Lo A.C., Shah D., Armendáriz M., Vannelli A., Mercaldo V., Trusel M., Li K.W., Gastaldo D., Santos A.R. (2019). The autism- and schizophrenia-associated protein CYFIP1 regulates bilateral brain connectivity and behaviour. Nat. Commun..

[128] Nir A., Barak B. (2021). White matter alterations in Williams syndrome related to behavioral and motor impairments. Glia.

[129] Morris C.A. (2010). Introduction: Williams syndrome. Am. J. Med. Genet. Part C Semin. Med. Genet..

[130] Osório A., Soares J.M., Prieto M.F., Vasconcelos C., Fernandes C., Sousa S., Carracedo Á., Gonçalves Ó.F., Sampaio A. (2014). Cerebral and cerebellar MRI volumes in Williams syndrome. Res. Dev. Disabil..

[131] Marie C., Clavairoly A., Frah M., Hmidan H., Yan J., Zhao C., Van Steenwinckel J., Daveau R., Zalc B., Hassan B. (2018). Oligodendrocyte precursor survival and differentiation requires chromatin remodeling by Chd7 and Chd8. Proc. Natl. Acad. Sci. USA.

[132] Bernier R., Golzio C., Xiong B., Stessman H.A., Coe B.P., Penn O., Witherspoon K., Gerdts J., Baker C., Vulto-Van Silfhout A.T. (2014). Disruptive CHD8 mutations define a subtype of autism early in development. Cell.

[133] Zhao C., Dong C., Frah M., Deng Y., Marie C., Zhang F., Xu L., Ma Z., Dong X., Lin Y. (2018). Dual requirement of CHD8 for chromatin landscape establishment and histone methyltransferase recruitment to promote CNS myelination and repair. Dev. Cell.

[134] Platt R.J., Zhou Y., Slaymaker I.M., Shetty A.S., Weisbach N.R., Kim J.A., Sharma J., Desai M., Sood S., Kempton H.R. (2017). Chd8 mutation leads to autistic-like behaviors and impaired striatal circuits. Cell Rep..

[135] Cotney J., Muhle R.A., Sanders S.J., Liu L., Willsey A.J., Niu W., Liu W., Klei L., Lei J., Yin J. (2015). The autism-associated chromatin modifier CHD8 regulates other autism risk genes during human neurodevelopment. Nat. Commun..

[136] Xu Q., Liu Y.Y., Wang X., Tan G.H., Li H.P., Hulbert S.W., Li C.Y., Hu C.C., Xiong Z.Q., Xu X. (2018). Autism-associated CHD8 deficiency impairs axon development and migration of cortical neurons. Mol. Autism.

[137] Galvez-Contreras A.Y., Campos-Ordonez T., Gonzalez-Castaneda R.E., Gonzalez-Perez O. (2017). Alterations of growth factors in autism and attention-deficit/hyperactivity disorder. Front. Psychiatry.

[138] Baumann N., Pham-Dinh D. (2001). Biology of oligodendrocyte and myelin in the mammalian central nervous system. Physiol. Rev..

[139] Hsieh J., Aimone J.B., Kaspar B.K., Kuwabara T., Nakashima K., Gage F.H. (2004). IGF-I instructs multipotent adult neural progenitor cells to become oligodendrocytes. J. Cell Biol..

[140] Beck K.D., Powell-Braxtont L., Widmer H.R., Valverde J., Hefti F. (1995). Igf1 gene disruption results in reduced brain size, CNS hypomyelination, and loss of hippocampal granule and striatal parvalbumin-containing neurons. Neuron.

[141] Ye P., Li L., Richards R.G., DiAugustine R.P., D’Ercole A.J. (2002). Myelination is altered in insulin-like growth factor-I null mutant mice. J. Neurosci..

[142] Steinman G. (2015). Plausible etiology of brain dysconnectivity in autism—Review and prospectus. Med. Hypotheses.

[143] Bozdagi O., Tavassoli T., Buxbaum J.D. (2013). Insulin-like growth factor-1 rescues synaptic and motor deficits in a mouse model of autism and developmental delay. Mol. Autism.

[144] Gonzalez-Perez O., Romero-Rodriguez R., Soriano-Navarro M., Garcia-Verdugo J.M., Alvarez-Buylla A. (2009). Epidermal growth factor induces the progeny of subventricular zone type B cells to migrate and differentiate into oligodendrocytes. Stem Cells.

[145] Gonzalez-Perez O., Quiñones-Hinojosa A. (2010). Dose-dependent effect of EGF on migration and differentiation of adult subventricular zone astrocytes. Glia.

[146] Gonzalez-Perez O., Alvarez-Buylla A. (2011). Oligodendrogenesis in the subventricular zone and the role of epidermal growth factor. Brain Res. Rev..

[147] Galvez-Contreras A.Y., Quiñones-Hinojosa A., Gonzalez-Perez O. (2013). The role of EGFR and ErbB family related proteins in the oligodendrocyte specification in germinal niches of the adult mammalian brain. Front. Cell. Neurosci..

[148] Russo A.J. (2014). Increased epidermal growth factor receptor (EGFR) associated with hepatocyte growth factor (HGF) and symptom severity in children with autism spectrum disorders (ASDs). J. Cent. Nerv. Syst. Dis..

[149] Kaphzan H., Hernandez P., Jung J.I., Cowansage K.K., Deinhardt K., Chao M.V., Abel T., Klann E. (2012). Reversal of impaired hippocampal long-term potentiation and contextual fear memory deficits in angelman syndrome model mice by ErbB inhibitors. Biol. Psychiatry.

[150] Chen J., Alberts I., Li X. (2014). Dysregulation of the IGF-I/PI3K/AKT/mTOR signaling pathway in autism spectrum disorders. Int. J. Dev. Neurosci..

[151] Onore C., Yang H., Van de Water J., Ashwood P. (2017). Dynamic Akt/mTOR signaling in children with autism spectrum disorder. Front. Pediatr..

[152] Cuscó I., Medrano A., Gener B., Vilardell M., Gallastegui F., Villa O., González E., Rodríguez-Santiago B., Vilella E., Del Campo M. (2009). Autism-specific copy number variants further implicate the phosphatidylinositol signaling pathway and the glutamatergic synapse in the etiology of the disorder. Hum. Mol. Genet..

[153] Zhou J., Blundell J., Ogawa S., Kwon C.H., Zhang W., Sinton C., Powell C.M., Parada L.F. (2009). Pharmacological inhibition of mTORCl suppresses anatomical, cellular, and behavioral abnormalities in neural-specific PTEN knock-out mice. J. Neurosci..

[154] Ness J.K., Mitchell N.E., Wood T.L. (2002). IGF-I and NT-3 signaling pathways in developing oligodendrocytes: Differential regulation and activation of receptors and the downstream effector akt. Dev. Neurosci..

[155] Butler M.G., Dazouki M.J., Zhou X.P., Talebizadeh Z., Brown M., Takahashi T.N., Miles J.H., Wang C.H., Stratton R., Pilarski R. (2005). Subset of individuals with autism spectrum disorders and extreme macrocephaly associated with germline PTEN tumour suppressor gene mutations. J. Med. Genet..

[156] Tilot A.K., Gaugler M.K., Yu Q., Romigh T., Yu W., Miller R.H., Frazier T.W., Eng C. (2014). Germline disruption of Pten localization causes enhanced sex-dependent social motivation and increased glial production. Hum. Mol. Genet..

[157] Frazier T.W., Embacher R., Tilot A.K., Koenig K., Mester J., Eng C. (2015). Molecular and phenotypic abnormalities in individuals with germline heterozygous PTEN mutations and autism. Mol. Psychiatry.

[158] Jones J.I., Clemmons D.R. (1995). Insulin-like growth factors and their binding proteins: Biological actions. Endocr. Rev..

[159] Linggi B., Carpenter G. (2006). ErbB receptors: New insights on mechanisms and biology. Trends Cell Biol..

[160] Galvez-Contreras A.Y., Campos-Ordonez T., Lopez-Virgen V., Gomez-Plascencia J., Ramos-Zuniga R., Gonzalez-Perez O. (2016). Growth factors as clinical biomarkers of prognosis and diagnosis in psychiatric disorders. Cytokine Growth Factor Rev..

[161] Furusho M., Dupree J.L., Nave K.A., Bansal R. (2012). Fibroblast growth factor receptor signaling in oligodendrocytes regulates myelin sheath thickness. J. Neurosci..

[162] Ishii A., Furusho M., Bansal R. (2013). Sustained activation of ERK1/2 MAPK in oligodendrocytes and schwann cells enhances myelin growth and stimulates oligodendrocyte progenitor expansion. J. Neurosci..

[163] Ishii A., Fyffe-Maricich S.L., Furusho M., Miller R.H., Bansal R. (2012). ERK1/ERK2 MAPK signaling is required to increase myelin thickness independent of oligodendrocyte differentiation and initiation of myelination. J. Neurosci..

[164] Bibollet-Bahena O., Almazan G. (2009). IGF-1-stimulated protein synthesis in oligodendrocyte progenitors requires PI3K/mTOR/Akt and MEK/ERK pathways. J. Neurochem..

[165] Cui Q.L., Almazan G. (2007). IGF-I-induced oligodendrocyte progenitor proliferation requires PI3K/Akt, MEK/ERK, and Src-like tyrosine kinases. J. Neurochem..

